# Musical Feature Processing and Learning Difficulties in Greek School-Aged Children: Piloting of a Screening Tool for Amusia

**DOI:** 10.3390/children13091156

**Published:** 2026-08-28

**Authors:** Sofia Manika, Nikolaos Trimmis, Ioannis Papakyritsis

**Affiliations:** Department of Speech & Language Therapy, University of Patras, 26504 Rio, Greece; papakyritsis@upatras.gr

**Keywords:** amusia, screening test, melodies, learning difficulties

## Abstract

**Highlights:**

**What are the main findings?**

- A high rate (59%) of suspected amusia was identified in children with learning difficulties compared to typical peers (8%).
- Demographic and background factors, including sex and music education, do not significantly impact performance of the music processing evaluation, whereas age and type of learning disorder did.

**What are the implications of the main findings?**

- The 15 min acoustic battery provides an efficient, quick screening tool for identifying children at risk for amusia in educational and clinical settings.
- Detecting amusia in children with learning difficulties enables targeted multidisciplinary interventions for auditory-musical processing deficits.

**Abstract:**

**Background/Objectives:** The present study aimed to describe a novel screening test for amusia and compare test performance between Greek children with learning difficulties and typical school-aged children. Additionally, it examined whether variables such as sex, age, and formal music education affect musical processing skills. **Methods:** We describe the construction and pilot testing of a musical processing screening tool consisting of seven tasks tapping on discreet auditory processing processes: “dissonant intervals”, “out of tone”, “interval discrimination”, “musical semantic memory”, “rhythm”, “melodic completion”, and “emotion”. The study sample included 200 participants: 100 typical students and 100 students with learning difficulties. **Results:** As expected, only 8% of typical students scored low, compared to 59% of the students with learning difficulties, indicating a possible relationship between the two conditions. Furthermore, factors such as gender and music education did not significantly affect musical processing performance, whereas age and type of learning disorder did. **Conclusions:** This condensed screening tool holds high practical and scientific value for efficiently identifying amusia deficits. It offers a viable comparative assessment for clinical and educational settings and can be effectively applied to both pediatric and general populations.

## 1. Introduction

Music and language perception are considered innate human capacities, with auditory processing of rhythm and melody beginning as early as the prenatal period [1,2]. While typical development leads to sophisticated musical awareness, a percentage of the population exhibits congenital amusia—a neurological deficit in pitch processing that persists despite normal hearing and intelligence [3].

Congenital amusia is traditionally defined as a neurodevelopmental deficit in fine-grained musical pitch perception, affecting approximately 1.5% to 4% of the general population [4,5]. While early investigations viewed amusia primarily as a domain-specific auditory impairment restricted to musical contexts, subsequent research has demonstrated broader implications across speech, prosody, and linguistic processing [6]. In particular, subtle pitch-processing deficits can disrupt acoustic tracking and spectral resolution, which are crucial for segmenting spoken language and acquiring phonological skills [7].

These early linguistic and phonological vulnerabilities often serve as core cognitive precursors not only for specific reading impairments (e.g., developmental dyslexia), but also cascade into broader academic underachievement and generalized learning difficulties across the curriculum.

A growing body of literature indicates a strong neuro-functional overlap between pitch/rhythmic auditory perception and learning difficulties, including developmental dyslexia [8,9]. According to the temporal sampling framework, impaired perception of auditory rise time and fine pitch variations negatively impact phonological awareness, speech-in-noise perception, and reading acquisition in primary school children [10]. Congenital amusia is not merely a “musical” problem; it is linked to broader neurological functions and can significantly overlap with learning and language difficulties [11]. Furthermore, recent findings highlight that amusic individuals exhibit impaired sensory consonance/dissonance evaluation, failing to perceive discordant or “unpleasant” musical intervals due to low-level sensory roughness processing deficits [12].

Despite these documented links between auditory perception and developmental learning challenges, standard clinical diagnostic batteries for amusia—such as the Montreal Battery for Evaluation of Amusia (MBEA)—were originally standardized on adult populations and often require lengthy testing sessions (45–60 min) with high cognitive and working memory demands [13]. While a shortened child-adapted version—the Montreal Battery of Evaluation of Musical Abilities [14]—has since been developed and validated for different age groups and cultural background, it was not specifically designed to assess comorbidity with language-based learning difficulties, nor does it includes subtests targeting harmonic integration. Consequently, there is a critical need for rapid, child-friendly screening instruments capable of identifying pitch discrimination vulnerabilities in primary school settings without imposing high working-memory loads.

Previous research has already established the presence of pitch-processing deficits and amusia in student populations using traditional, full-scale diagnostic batteries. However, administering these long-form tools in school environments presents practical challenges, as lengthy protocols often induce attentional fatigue and task disengagement in young learners. To address this gap, the present research introduces a new, condensed 15 min acoustic screening battery based on the diagnostic principles of the Montreal protocol. Crucially, this tool is not designed to establish a conclusive clinical diagnosis of amusia; rather, it functions as an additional, fast-track screening instrument specifically engineered to flag suspected amusia and pitch-processing vulnerabilities in students. Utilizing piano-timbred stimuli, the battery evaluates seven key musical acoustic dimensions: dissonant intervals, out of tone, interval discrimination, rhythm, musical semantic memory, emotion, and completion. Specifically, this study pursues two complementary objectives: (1) to evaluate whether a shortened 15 min acoustic battery assessing musical feature processing can effectively differentiate typical students from those with learning difficulties, thereby serving as a practical screening alternative to existing lengthier batteries; and (2) to use this tool to examine the extent to which amusia co-occurs with learning difficulties, and whether demographic factors such as age, sex, and prior music education influence screening outcomes. Finally, this study investigates whether these newly introduced subtests assessing dissonant interval recognition and melodic completion are essential and clinically relevant for screening for music processing difficulties.

### The Amusic Profile: Characteristics, Diagnosis, and Identification

The term amusia was first coined in 1871 by Steinthal, who explored the disorder as an inability in musical processing [15]. Subsequently, in 1888, Knoblauch framed the disorder as an impairment in processing, understanding, producing, reading, or writing music [16]. Since then, significant research has been conducted to delineate this condition. Specifically, amusia is defined as a neurological disorder characterized by deficits in acoustic tone discrimination [7,8], the memory of familiar melodies, and frequently, the rhythmic perception of auditory stimuli [3,13].

Depending on its etiology, the onset of amusia is classified into two primary types: congenital and acquired [10]. Congenital amusia is present from birth, stemming from subtle neurodevelopmental anomalies in brain connectivity [4,17]. Conversely, acquired amusia results from brain damage sustained later in life, occurring in individuals who previously possessed a fully functional music processing system [18,19]. While both types occur in the presence of normal cognitive functions and peripheral hearing, they manifest with highly similar core deficits, such as impaired harmonic sequence detection [20].

In daily life, this disorder manifests as a significant difficulty in musical perception and the acquisition of basic musical skills [12]. Those affected are typically unable to differentiate between melodies that differ by only a few notes or a semitone [21,22]. Beyond simple pitch discrimination, individuals with amusia often exhibit secondary deficits in emotional responses to music, as they struggle to capture the affective nuance of melodies [23]. Crucially, this musical disability occurs independently of peripheral auditory processing dysfunction, lack of music education, or general intellectual deficits [3].

From a cognitive perspective, this profile frequently exhibits a high comorbidity with learning challenges, particularly developmental dyslexia. Because music and language processing rely on overlapping neural resources, the deficits in phonology and reading found in dyslexia often share a common cognitive denominator with the pitch discrimination impairments seen in “tone deafness” [18].

Given this significant overlap between auditory processing deficits and learning difficulties, early identification is crucial within educational and clinical contexts. However, established full-length diagnostic batteries (e.g., the 180-trial MBEA) require lengthy testing sessions that often induce severe cognitive and attentional fatigue in primary school children—particularly those with learning difficulties—thereby compromising response validity. To overcome these limitations, the present study introduces a targeted, rapid (15 min) screening tool specifically optimized for pediatric populations. Rather than replacing a full diagnostic battery, this screening tool aims to efficiently flag amusia risk co-occurring with learning challenges, while evaluating whether two novel behavioral tasks—dissonant intervals and melody completion—provide clinically relevant diagnostic indicators for rapid screening.

## 2. Materials and Methods

The present study describes a novel, 15 min quantitative screening battery designed to evaluate musical processing across seven acoustic dimensions: interval dissonance, out-of-tune detection, melodic interval discrimination, rhythm, musical semantic memory, emotional perception of musical elements, and melodic completion. The screening test is implemented on a pilot basis in two groups of primary school children: typically developing children and children with learning difficulties.

### 2.1. Participants

The sample consisted of 200 primary school students aged 6 to 12 years old, divided into two distinct groups: 100 neurotypical controls and 100 students with learning difficulties. The control group was essential to (a) determine an empirical normative baseline from which the diagnostic screening threshold (M-2SD = 29.4 ≈ 30) could be established, and (b) evaluate the discriminant validity of the novel screening tool by directly comparing pitch-processing performance between groups. The control group (*N* = 100) included children displaying typical-to-high academic performance based on official school records and teacher evaluations. To ensure the sample was representative of the general neurotypical pediatric population, the following inclusion criteria were set: (a) the complete absence of any formal clinical diagnosis of learning, language, or neurological disorders, (b) no history of speech-language therapy, and (c) confirmed normal hearing thresholds (≤15 dB HL). Recruited from mainstream educational settings, this group provides a robust baseline of typical developmental performance.

The experimental group (*N* = 100) had a formal clinical diagnosis of learning difficulties obtained from certified public diagnostic centers (70%, *N* = 70), including developmental dyslexia (*N* = 10), reading disorders (*N* = 14), spelling deficits (*N* = 9), dyscalculia (*N* = 2), and mixed generalized learning disorders (*N* = 35, operationally defined as students exhibiting persistent academic impairments across multiple core domains rather than a single isolated deficit). The remaining 30% (*N* = 30) consisted of undiagnosed students exhibiting low academic performance, below the standardized cut-off threshold, confirmed via the AMDE [24], a standardized Greek screening tool for learning difficulties administered by classroom teachers and verified by a certified speech-language therapist. Specifically, the AMDE evaluates students across six core cognitive and academic domains (receptive language, expressive language, reading, writing, mathematics, and reasoning), capturing a broad educational vulnerability profile that confirms their classification into generalized learning difficulties.

All participants (*N* = 200) presented normal hearing thresholds (≤15 dB HL) and had no uncorrected sensory deficits. The sample had an even sex distribution, including 102 boys (51%) and 98 girls (49%). Regarding musical background, 77% of the sample had received no formal music instruction, while 23% had at least two years of music training. Written informed consent was formally obtained from the parents or legal guardians of all participating children prior to testing.

### 2.2. Test Development: Task Architecture and Stimuli Composition

While the core framework of the battery is modeled after the Montreal Battery for the Evaluation of Amusia (MBEA; [13]), this tool incorporates two specialized components, dissonant interval recognition and melodic completion, to address secondary processing deficits identified in the recent literature. The battery includes seven subtests, each consisting of six different items (yielding a total of 42 scored items). All experimental musical stimuli were initially composed and prepared using the music notation software MuseScore (Muse Group, Limassol, Cyprus). All melodies were recorded in Ioannina, Greece, at a tempo of 80 BPM, using a Rode USB microphone (Rode Microphones, Sydney, Australia). Afterwards, participants receive explicit instructions directing their attention toward specific acoustic features during listening. Child-friendly and simplified verbal prompts were utilized across all subtests to ensure full comprehension by primary school children regardless of prior musical education. The auditory inventory included 12 musical interval pairs (dissonant intervals Task) and 55 individual melodies, distributed across the seven subtests. Each individual task is described in detail in the following section.

#### 2.2.1. Dissonant Intervals

**Figure 1:**
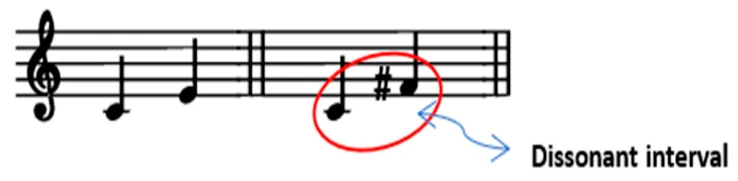
Visual representation of dissonant vs. consonant interval used in the screening test.

While the standard Montreal Battery of Evaluation of Amusia (MBEA) provides a comprehensive assessment, its full administration across 180 trials is time-consuming and can induce participant fatigue. To optimize diagnostic efficiency and sensitivity, this targeted ‘Dissonant Intervals’ subtest was introduced to isolate fine dissonance discrimination, thereby substantially reducing testing duration while maintaining high diagnostic accuracy. From an acoustic perspective, the dissonant intervals examined date back to Pythagorean tuning principles and include minor/major seconds, semitones, and augmented fourths (tritones) [25]. These specific stimuli are typically perceived by neurotypical listeners as “discordant” or “incorrect” [26]. Specifically, this subtest evaluates interval dissonance using 6 items, each including two paired stimuli (12 interval pairs total; 6 responses) to identify off-pitch tones. Prior to test administration, children received standardized verbal instruction and they were asked to identify which one of the musical sounds in each pair sounded unfamiliar to human ear, with responses recorded as either correct or incorrect. The selected “discordant” intervals include C-F# (Figure 1), D-G#, G b-A, C+-D#, G#-C+, and F#-G. For each item the listener is asked to identify which of the two notes is unfamiliar.

#### 2.2.2. Out of Tone

**Figure 2:**
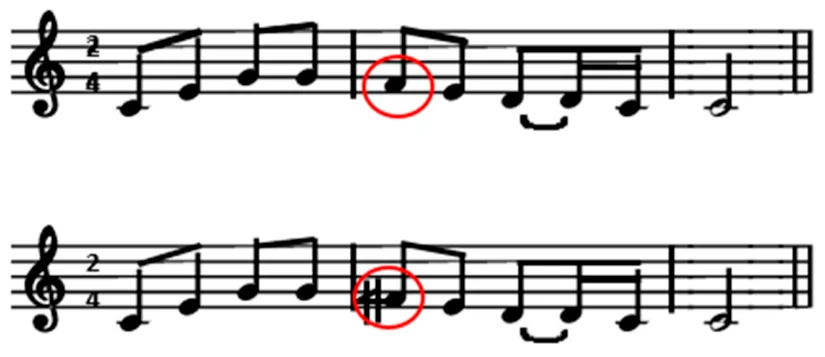
Illustration of the “out of tone’’ task, where two melodies differ by a single note to assess auditory discrimination of proximal frequencies and tonal violations.

A primary diagnostic indicator of amusia is a profound difficulty in identifying notes that deviate from the distance of tone or semitone. In this subtest, subjects are required to compare melodic pairs that differ by only a single pitch [27]. Six pairs of melodies were composed based on Western classical harmonic principles, utilizing the scales of C major, Eb major, and F major. The critical pitch alteration is strategically positioned on a strong beat within either the final or penultimate measure of the sequence. These nearly identical melodies introduce a “foreign” or non-diatonic note, typically displaced by a single semitone (Figure 2) or whole tone from the original pitch—a nuance that amusic individuals generally fail to detect. To maintain participant engagement and ensure sustained auditory attention, one pair consisting of perfectly identical melodies is included in the set. This task evaluates accuracy of pitch discrimination using six pairs of minimally different melody items (12 melodies total; 6 responses). This subtest evaluated the participant’s ability to discriminate between the different melodies. Responses were scored on a binary scale (correct/incorrect).

#### 2.2.3. Interval Discrimination

This subtest assesses interval discrimination by introducing a subtle semitone-level pitch alteration between two otherwise structurally identical melodies, while the overall melodic contour remains unchanged.

**Figure 3:**
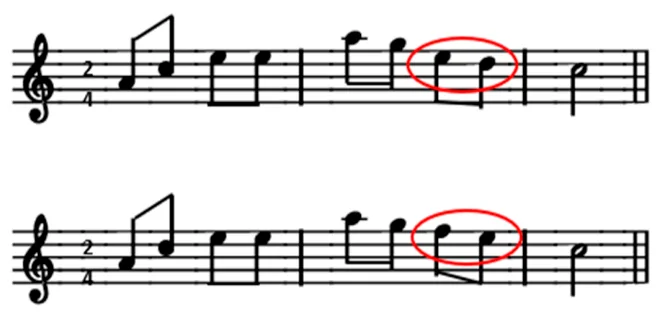
Example of the interval discrimination subtest stimuli, featuring two melodies that differ in pitch direction. The red circles indicate the notes that differ by a semitone and a tone, respectively).

Unlike the ‘out-of-tune’ task, which involves an explicit tonal violation (an out-of-key note), the Interval Discrimination subtest utilizes key-congruent alterations that sound musically familiar. Consequently, the inability to distinguish between these melodies stems from a deficit in fine-grained pitch discrimination, short-term melodic memory, or a combination of both [28]. While the former relates to the auditory system’s failure to detect frequency differences, the latter involves the participant’s inability to retain the melodic structure in memory long enough for comparison. Consequently, this experimental methodology aims to identify amusia by isolating whether the diagnostic failure stems from sensory pitch perception or cognitive remembrance of melodic shifts [29]. This task measures pitch direction sensitivity using six items consisting of pairs of melodies (12 melodies total; 6 responses). Regardless of whether the melodic variation is harmonically “correct” or “out of tone”, amusic individuals are typically unable to discern these subtle shifts. This subtest evaluated participants’ ability to discriminate whether the two melodies in each pair are identical or different (Figure 3), with responses scored on a binary scale (correct/incorrect).

#### 2.2.4. Musical Semantic Memory

This experimental task is implemented to recognize familiar melodies. A failure in recognition stems from a fundamental deficit in melodic memory, which is largely representative of underlying difficulties in pitch perception. It is critical to note that amusic individuals struggle to differentiate between structurally similar musical sequences due to impaired acoustic pitch accuracy, a factor that subsequently hinders their long-term retrieval of musical information [30]. To assess these mnemonic functions, participants are presented with several culturally prominent melodies to determine if they can successfully identify well known melodies when they are presented without lyrics.

**Figure 4:**
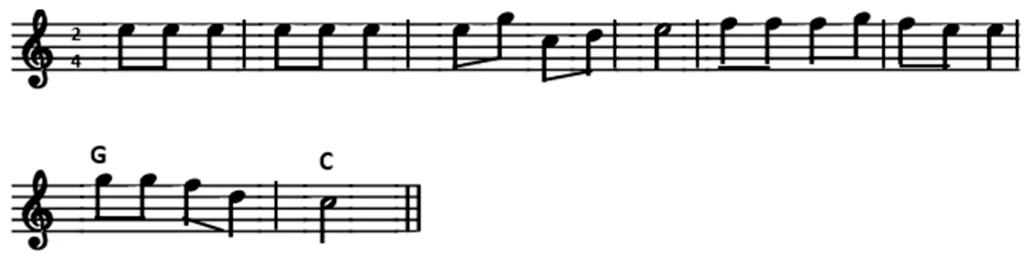
Familiar instrumental melody to evaluate melodic recognition.

This task assesses long-term melodic memory across six single-melody items aimed at identifying familiar tunes (6 melodies total; 6 responses). For the Musical Memory subtest, participants listened to instrumental (lyrics-free) excerpts of highly familiar melodies and were asked: ‘Do you know this melody?’ Responses were recorded on a binary scale (Yes/No). More specifically, the selection of stimuli for this assessment includes widely recognized pieces such as “Twinkle, Twinkle, Little Star” (Figure 4), “Bella Ciao”, “The Little Rooster”, “Jingle Bells”, “Rudolph the Red-Nosed Reindeer”, and “The Drummer”. These pieces are widely used in public preschool and school settings in Greece.

#### 2.2.5. Rhythm Perception

Beyond tonal deficits, a subset of the amusic population exhibits significant impairments in the perception of musical rhythm. This condition is fundamentally linked to broader temporal processing mechanisms, specifically the listener’s ability to differentiate the intensity and duration of auditory stimuli. Research suggests that diminished rhythmic perception serves as a clinical marker for deficits in rapid temporal auditory processing. Furthermore, this characteristic provides an indirect link to dyslexia, highlighting a notable comorbidity between amusia and various learning difficulties. Specifically, this subtest assesses rhythmic discrimination across 6 items consisting of melodies with subtle temporal variations (12 melodies total; 6 responses). In the initial items, the acoustic stimuli are differentiated rhythmically by altering the duration of only two notes within a single meter, while subsequent items utilize pairs of complete melodies to assess rhythmic variances. Participants were prompted with the question: ‘’Is the second melody faster, slower, or at the same speed as the first?’’, with responses recorded accordingly.

#### 2.2.6. Completion

In neurotypical listeners, the perception of melodic completion relies on the ability to encode fine-grained pitch intervals and construct implicit mental representations of tonal syntax. Through lifelong exposure to music, typical listeners automatically project melodic expectations and sense tonal closure when a phrase resolves to a stable pitch, such as the tonic [31,32]. Conversely, individuals with congenital amusia exhibit a selective impairment in fine pitch processing that severely disrupts their ability to evaluate melodic resolution and closure [3,13]. While amusic individuals may passively track pitch probabilities at an implicit level, they fail to translate this knowledge into conscious, explicit judgments regarding melodic completion—a deficit broadly attributed to a breakdown in fronto-temporal connectivity [33,34,35]. Following methodologies that evaluate completion ability by truncating musical excerpts at strategic points [36], this task assesses the explicit recognition of tonal resolution.

**Figure 5:**
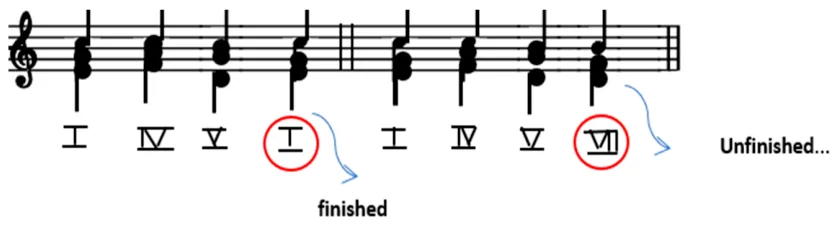
Musical notation illustrating the Melodic Completion task.

Specifically, this subtest evaluates structural expectation and melodic completion across 6 trials (7 melodies total; 6 responses). The first trial presents a comparative pair of melodies constructed according to Western harmonic principles: the first provides full resolution to the tonic, whereas the second terminates on the leading tone (seventh degree) to evoke non-completion (Figure 5). The remaining five trials present individual musical excerpts from standard repertoire. Three phrases provide complete tonal closure resolving to the tonic (including selections from Heller’s Etude op. 46 No. 7 in E minor, Bach’s Concerto in D minor, and the folk melody “Inside this boat”), whereas two excerpts are prematurely interrupted at the third harmonic tier (specifically a variation from Heller’s Etude and an arrangement of Linkin Park’s “Numb”). Participants were prompted with the standardized question: “Does the melody finish correctly?”, with responses scored on a binary scale (correct/incorrect).

#### 2.2.7. Emotion

In addition to deficits in structural integration, congenital amusia is often associated with a secondary impairment in the perception of emotional qualities conveyed by musical melodies. This difficulty in decoding the expressive intent of a composition suggests a breakdown in the processing of affective auditory stimuli [37]. Specifically, this subtest assesses emotional perception across six single-melody trials (6 melodies total; 6 responses). Participants are required to categorize each according to its perceived emotional valence, specifically distinguishing between “happy” and “sad” characteristics, and they were prompted with the question: “Which emotion does the melody evoke?”, with each response was scored as either correct in incorrect.

The experimental stimuli are categorized as follows:

- Positive Emotional Valence (Happy): The selection includes Mozart’s Sonata Allegro No. 279 and Carlos Gardel’s waltz, Por una Cabeza.
- Negative Emotional Valence (Sad): For the evaluation of melancholic expressiveness, the study employs Beethoven’s Moonlight Adagio sostenuto, L. Machairitsas’ Notos, Yann Tiersen’s The Piano, and The Train Leaves at Eight by Manos Eleftherios.

### 2.3. Procedure

All auditory stimuli were constructed in accordance with Western tonal-harmonic conventions, strictly utilizing diatonic major scales. The melodies were generated using a standardized piano timbre and recorded in uncompressed. wav format at a constant tempo of 80 beats per minute (bpm) using Adobe Audition 3. The evaluation was conducted in a controlled laboratory environment to ensure focus and minimize external auditory factors, with each session lasting approximately 15 min.

### 2.4. Scoring

As described above, the screening battery comprises seven subtests of six individual trials each, yielding a cumulative maximum score of 42 points (1 point per correct item and six points per subtest). Adopting the statistical framework of the MBEA, a baseline screening cutoff threshold was established at 29.4 out of 42 points (2 SD below the mean of typical peers). Scores falling below this threshold indicate suspected amusia (tone deafness), warranting further clinical diagnostic assessment via the full MBEA.

### 2.5. Statistical Analysis

Statistical analyses were performed using IBM SPSS Statistics (Version 28.0, IBM Corp., Armonk, NY, USA). Continuous variables were tested for normality and homogeneity of variance. Due to violations of the homogeneity of variance assumption (Levene’s test, *p* < 0.001), Welch’s t-tests (equal variances not assumed) were utilized for parametric group comparisons to provide robust test statistics. Categorical group distributions were evaluated using Chi-square tests of independence, reporting Cramér’s V for effect size. Multiple linear regression analysis was executed to evaluate predictors of overall performance while controlling for age, sex, and music education. Statistical significance was set at *p* < 0.05, and exact *p*-values, confidence intervals (95% CI), and effect sizes (Cohen’s d, R^2^, Cramér’s V) are reported throughout.

## 3. Results

### 3.1. Psychometric Evaluation and Reliability

Prior to evaluating group differences, the internal consistency of the 7-subtest screening battery was assessed across the total sample (*N* = 200). The analysis demonstrated good internal consistency (Cronbach’s a = 0.78), confirming that the subtests reliably measure a unified construct of auditory processing.

### 3.2. Overall Test Performance

**Figure 6:**
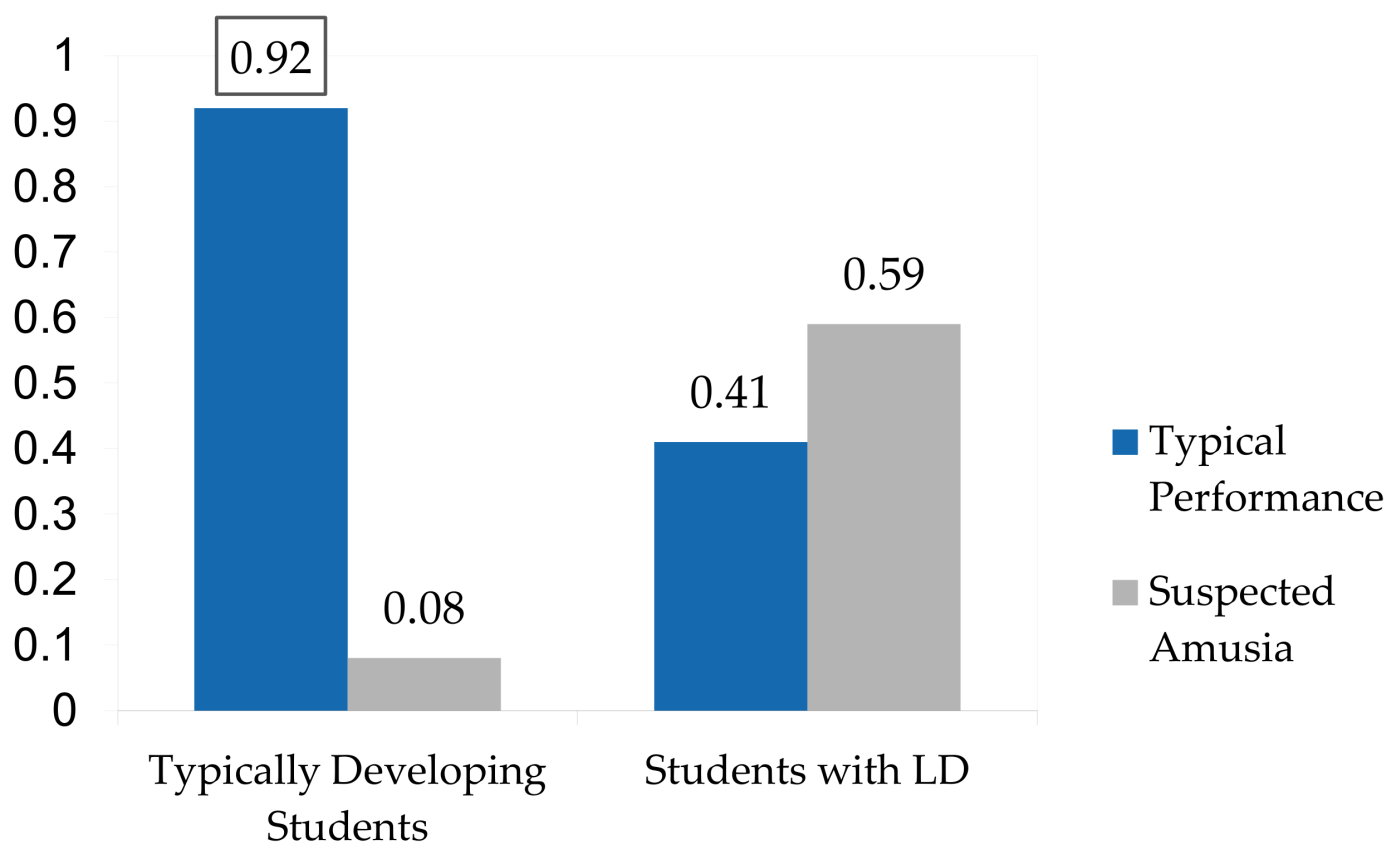
Comparison of amusia screening test performance between typically developing students and students with learning difficulties.

The evaluation process began with a descriptive analysis to determine the prevalence of suspected amusia within the study sample. As expected, a significant discrepancy was observed between the two groups. Specifically, 59% of the students with learning difficulties scored below the established baseline of 29.4, whereas only 8% of typical students presented a similar pattern. These findings, which illustrate the high co-occurrence of difficulties with musical processing and learning difficulties, are visually summarized in Figure 6.

Following the descriptive overview, an inferential statistical analysis was conducted to examine the strength of these associations. A chi-squared test confirmed that the presence of learning difficulties and performance on the amusia screening tool are significantly dependent variables, *χ*^2^(1, *N* = 200) = 58.38, *p* < 0.001, yielding a Cramér’s *V* = 0.54. To further refine these results, a regression analysis was utilized to control for potential confounding factors. While musical training and sex were found to have no significant effect on the outcomes, age emerged as a significant factor, positively influencing test performance. However, the presence of learning difficulties remained the most robust predictor of the overall results. A detailed breakdown of how different types of learning difficulties correlate with test failure is presented in Table 1.

The prevalence of amusia was not uniform across all learning difficulty categories. Interestingly, all students with a diagnosis of specific learning disorders scored below the designated cut-off point, whereas children with generalized LD and without a formal related diagnosis had higher performance. This breakdown clarifies the aggregate 59% rate observed in the clinical group (*n* = 100). In Table 1, *N* represents the total number of students per category, while the ‘Suspicion of amusia’ column indicates the number of individuals who scored below the 29.4 cutoff point. These findings align with the overall data presented in Figure 6.

Subsequently, a multiple regression analysis was conducted to examine which variables significantly predict performance on the screening tool (R^2^ = 0.299, adjusted R^2^ = 0.284, F(4, 195) = 20.766, *p* < 0.001). As shown in Table 2, sex (B = −1.200, SE = 0.839, β = −0.086, *p* = 0.154) and music education (B = 0.421, SE = 0.645, β = 0.041, *p* = 0.515) did not significantly predict test performance. In contrast, age emerged as a significant predictor, with increasing age associated with better performance (B = 0.887, SE = 0.245, β = 0.221, t = 3.622, *p* < 0.001, 95% CI [0.40, 1.37]). Most importantly, the presence of learning difficulties was the strongest negative predictor of performance (B = −6.336, SE = 0.869, β = −0.454, t = −7.291, *p* < 0.001, 95% CI [−8.05, −4.62]).

### 3.3. Subtest Profile Comparison Between Amusic and Non-Amusic Students

To further elucidate the specific cognitive loci of musical impairment, a detailed profile analysis was conducted across all seven subtests, comparing all students with suspected amusia (*n* = 67) to their non-amusic peers (*n* = 133). The mean scores and standard deviations for both groups are presented in Table 3.

As shown in Table 3, students who scored above the baseline consistently demonstrated markedly higher performance across all core pitch-based tasks. The most pronounced deficits in students with suspected amusia were observed in interval discrimination (Interval Discrimination: M = 2.27, SD = 1.24 vs. M = 4.08, SD = 0.64), tonal memory (Memory: M = 2.33, SD = 1.27 vs. M = 4.69, SD = 0.74), and tone discrimination (Tone: M = 2.70, SD = 1.27 vs. M = 4.51, SD = 0.67). Regarding the rhythm subtest, although amusic students exhibited lower scores (M = 3.90, SD = 1.16 vs. M = 5.42, SD = 0.84), their performance on rhythm was substantially higher relative to their fine-grained pitch perception and memory scores, confirming that pitch processing constitutes the primary locus of impairment in congenital amusia. Similarly, amusic participants displayed their highest relative performance in emotion recognition (Emotion: M = 4.19, SD = 1.43), scoring considerably higher than in fine tonal discrimination tasks (out of tone & interval discrimination tasks). Finally, performance in the newly introduced subtests, dissonant interval, which examines fine dissonance discrimination skills, and musical completion, which assesses melodic integration, was in the mid-range (M = 3.10, SD = 1.33 for completion, M = 3.07, SD = 1.06 for dissonant interval). Overall, these findings indicate that while fine acoustic pitch discrimination is substantially compromised in amusic individuals, high-order affective processing and rhythm perception remain relatively better preserved.

### 3.4. Bivariate Correlation and Scatterplot Matrix Analysis

**Figure 7:**
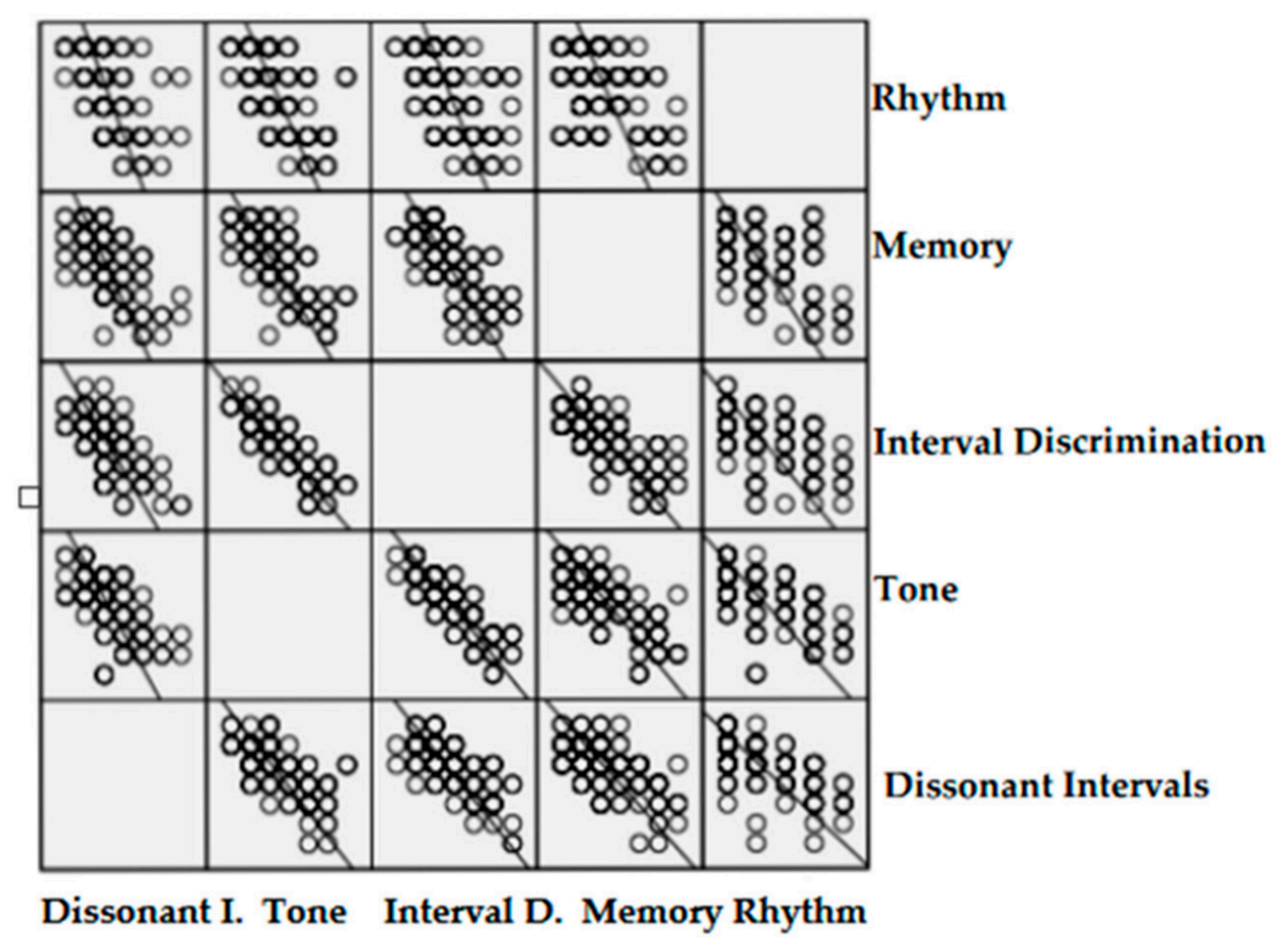
Scatterplot matrix illustrating the pairwise relationships among performance scores in pitch perception (Dissonant Interval, Tone, Interval Discrimination), tonal memory (Memory), and rhythmic perception (Rhythm) subtests.

To evaluate the interrelationships among the acoustic and cognitive subtests, a scatterplot matrix was generated for the core pitch-processing (Dissonant Interval, Out of Tone, Interval Discrimination, Musical Semantic Memory) and the rhythm task, as shown in Figure 7.

Pitch Processing and Tonal Memory Interrelationships: A robust positive linear trend is visually evident across the pitch-based subtests and musical semantic memory. The tight clustering of data points along the positive diagonal axis indicates strong positive correlations among Out of Tone, Interval Discrimination, Dissonant Interval, and Memory. Participants who demonstrated high proficiency in fine-grained acoustic pitch discrimination consistently exhibited superior performance in tonal memory and interval perception tasks.

Rhythmic Perception Dissociation: In contrast, the bivariate distributions involving the Rhythm subtest display a broader dispersion of data points relative to the pitch-related variables. While maintaining a general positive association with overall performance, the distinct scatter pattern of the Rhythm task reflects a relative cognitive dissociation between pitch/tonal processing and rhythmic perception, supporting the domain-specific nature of pitch deficits in auditory processing assessments.

**Figure 8:**
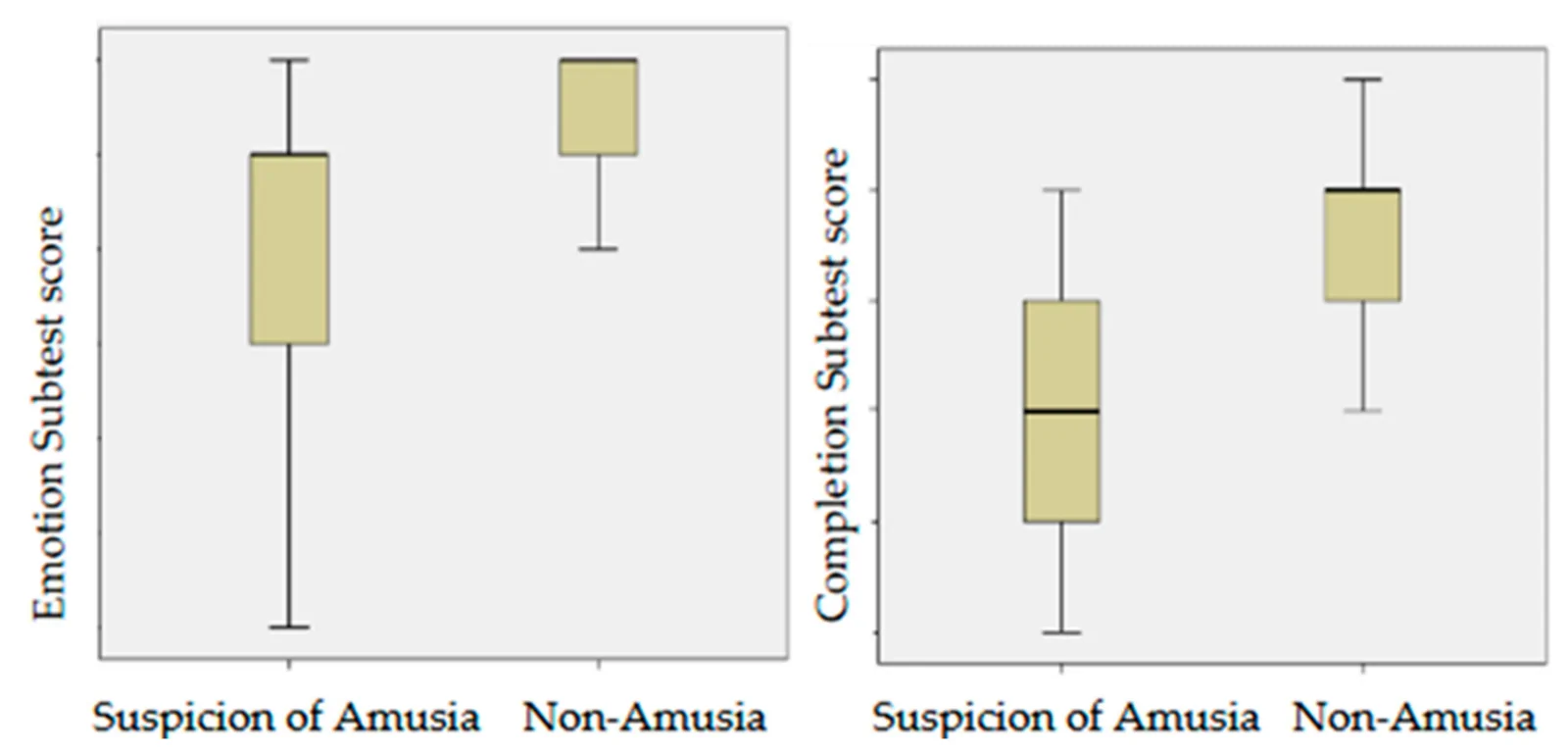
Boxplot distribution for the Emotion and Melodic Completion subtests. The central line represents the median score, highlighting the performance variance between the two groups.

The final stage of the analysis involved comparing groups across two of the test tasks: emotion and melodic completion. As illustrated in the boxplots (Figure 8), students with scores below the designated baseline demonstrated unexpectedly high scores in the emotional dimension of the test. Conversely, their performance on the completion subtest reached only moderate levels. This finding suggests that melodic integration ability may constitute a secondary deficit in amusia, a hypothesis that warrants further investigation. In conclusion, while the present screening tool effectively identifies suspected potential cases of amusia, a full diagnostic assessment remains the recommended next step to confirm a clinical diagnosis. To formally evaluate these differences, Welch’s *t*-tests were conducted. For the Emotion subtest, participants with suspected amusia scored significantly lower (M = 4.11, SD = 1.46, 95% CI [3.74, 4.49]) compared to the non-amusia group (M = 5.49, SD = 0.61, 95% CI [5.39, 5.59]), t(69.23) = −7.081, *p* < 0.001, Mean Diff = −1.37, Cohen’s d = 1.44. Although their scores in the emotional dimension remained unexpectedly high, the statistical difference was substantial. Conversely, on the completion subtest, performance in the amusia group reached only moderate levels (M = 2.98, SD = 1.32, 95% CI [2.64, 3.32]) compared to typical controls (M = 4.78, SD = 0.75, 95% CI [4.65, 4.91]), t(77.56) = −9.909, *p* < 0.001, Mean Diff = −1.79, Cohen’s d = 2.01. In conclusion, while the present screening tool effectively identifies potential cases of amusia, a full diagnostic assessment remains the recommended next step to confirm a clinical diagnosis.

## 4. Discussion

The present study evaluated the effectiveness of a novel, condensed 15 min acoustic screening tool for identifying possible deficits in the processing of musical features, a possible sign of congenital amusia, a neurodevelopmental disorder in school-aged children. By integrating the traditional dimensions of the Montreal Battery of Evaluation of Amusia (MBEA) with two novel subtests, this research provides preliminary data for the validation of a practical and time-efficient alternative for to large-scale clinical and educational assessments. Rather than serving as a definitive diagnostic tool or re-proving the existence of comorbidity between amusia and learning difficulties (LD), the primary utility of this battery lies in its ability to rapidly flag pitch-processing risks in pediatric populations without inducing severe cognitive fatigue.

A central contribution of this battery is the inclusion of two novel subtests designed to complement standard pitch discrimination tasks:

- Diagnostic Utility of “Dissonant Intervals”: This subtest was introduced to directly target the core deficit of congenital amusia—specifically, the impaired processing of fine pitch variations and sensory roughness within harmonic conventions. Children with suspected amusia exhibited a marked drop in performance on this task, confirming that dissonance perception serves as a sensitive behavioral marker for pitch-processing impairments.
- Clinical and Pedagogical Utility of “Melodic Completion”: This task serves a dual purpose. Diagnostically, it assesses higher-level tonal syntax and auditory expectancy (the ability to predict phrase resolution). Methodologically, its playful and gamified nature (“playful nature”) utilizes complete melodic phrases rather than isolated tonal pairs. This significantly reduced cognitive fatigue and maintained high attentional engagement among primary school children with LD.

Our findings align with international literature, indicating an amusia suspicion rate of 8% among neurotypical controls and 59% among students with learning difficulties. Crucially, this observed rate in the LD cohort closely aligns with prevalence estimates reported in prior literature using full-scale diagnostic batteries. This empirical agreement supports the convergent validity of the condensed tool, proving that reducing administration time to 15 min does not compromise screening sensitivity. Rather than establishing novel comorbidity, these findings confirm established theoretical models—such as the “shared-resource” hypothesis and temporal sampling framework—suggesting that music and language processing rely on overlapping neural mechanisms (e.g., structural variations in the arcuate fasciculus).

### Limitations and Future Directions

Despite the promising diagnostic sensitivity and practical utility of the proposed screening tool, the following limitations should be noted. To protect primary school children with learning disabilities from severe attentional and cognitive fatigue, formal cross-validation against the full 180-trial MBEA protocol was not performed in this pilot trial. While the battery demonstrated robust discriminant validity between neurotypical controls and students with LD, future studies should conduct concurrent criterion-validation against established long-form batteries in controlled clinical settings. Additionally, although the “Melodic Completion” subtest elicited strong student involvement and was effective in preventing test disengagement, holistic and affective task may introduces confounding variance when isolating pure pitch deficits. Larger, fully powered samples are required to determine whether these subtests should remain core screening components or be categorized as supplementary measures of preserved affective music processing. Similarly, although key demographic covariates (age, sex, and formal music education) were controlled for in regression models, unmeasured domain-general cognitive factors—such as individual variations in working memory capacity and selective auditory attention—could influence performance on interval discrimination tasks. Finally, the current sample size did not allow for isolated sub-analyses across specific learning disability subtypes (e.g., dyscalculia-specific comorbidity). Given potential theoretical overlaps in spatial, temporal, and parietal processing, future research incorporating larger subgroup samples is needed to investigate these specific relationships further.

## 5. Conclusions

Overall, the findings of this study demonstrate the screening efficiency and practical utility of a novel, condensed 15 min acoustic battery for flagging pitch-processing risk in school-age populations. By offering a time-efficient and accessible behavioral assessment specifically tailored for children—including those with learning difficulties—this tool successfully bridges the gap between lengthy diagnostic protocols and feasible classroom or clinical screening. Furthermore, the melodic completion of these findings into music pedagogy and audiology provides valuable insights into auditory-cognitive mechanisms. By confirming the strong overlap between pitch-processing deficits and learning difficulties, this screening tool serves as a foundation for future research aimed at targeted pedagogical supports. Ultimately, early risk identification can inform tailored interventions to enhance acoustic accuracy and rhythmic perception across both musical and linguistic domains, fostering a more holistic educational approach for students with learning difficulties.

## Figures and Tables

**Table 1:** Prevalence of suspected amusia according to the type of Learning Difficulties.

Diagnosis	Non-Amusia	Suspicion of Amusia	Total
Dyslexia	0	10 (100%)	10
Reading Disorder	0	14 (100%)	14
Spelling Deficits	0	9 (100%)	9
Dyscalculia	0	2 (100%)	2
Generalized LD	16 (45.7)	19 (54.3%)	35
LD without diagnosis	25	5 (16.7%)	30
Total	41	59	100

Note: The failure rate refers to scores below the 29.4 baseline. LD = Learning Difficulties. The ‘LD without diagnosis’ category includes students identified through the AMDE (Screening Tool for Performance Difficulties), a validated Greek screening instrument used in school settings.

**Table 2:** Statistical analysis of regression about variables and score.

Model	*B*	*SE*	*t*	*p*
Constant	30.059	1.133	26.533	<0.001
Age	0.887	0.245	3.622	<0.001
Sex	−1.200	0.839	−1.430	0.154
Learning difficulties	−6.336	0.869	−7.291	<0.001
Music education	0.421	0.645	0.653	0.515

**Table 3:** Mean values and standard deviations (SD) of scores across all performance subtests for participants that performed above (non-amusic) and below (amusic) the established baseline.

Participants (*N*)	Dissonant Interval	Out of Tone	Interval Discrimination	Memory	Rhythm	Emotion	Completion
Non-Amusic (*n* = 133)	4.48 (0.66)	4.51 (0.67)	4.08 (0.64)	4.69 (0.74)	5.42 (0.84)	5.51 (0.60)	4.79 (0.76)
Amusic (*n* = 67)	3.07 (1.06)	2.70 (1.27)	2.27 (1.24)	2.33 (1.27)	3.90 (1.16)	4.19 (1.43)	3.10 (1.33)
Total (*N* = 200)	4.01 (1.05)	3.91 (1.25)	3.47 (1.23)	3.90 (1.47)	4.91 (1.20)	5.07 (1.14)	4.23 (1.27)

## Data Availability

The screening tool developed as part of this study is available online as Supplementary Materials (Files S1 and S2). The SPSS data supporting the findings of this study are available in Figshare at https://doi.org/10.6084/m9.figshare.29651258.v1. Moreover, all data and related materials can be provided by the first author on request.

## Supplementary Materials

The following supporting information can be downloaded at: https://www.mdpi.com/article/10.3390/children13091156/s1, File S1: Melodies of Amusia; File S2: Questionnaire Responses.
