# Supplementary material for: Musical Feature Processing and Learning Difficulties in Greek School-Aged Children: Piloting of a Screening Tool for Amusia
Source: Children (Basel). 2026 Aug 28;13(9):1156. doi: 10.3390/children13091156 (PMC13605103; doi:10.3390/children13091156)

M E L O D I E S

1. Dissonant Intervals

(a)

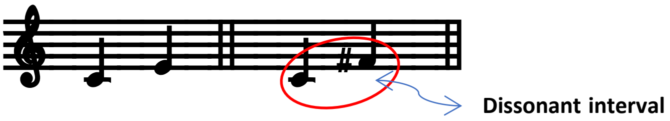

(b)

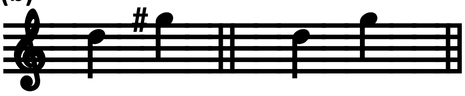

(c)

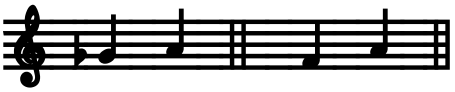

(d)

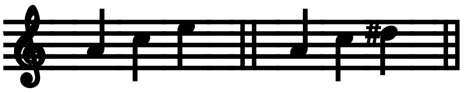

(e)

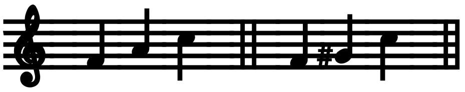

(f)

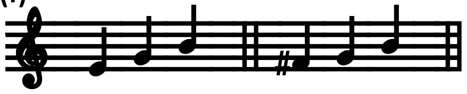

2. Out of tone

(a)

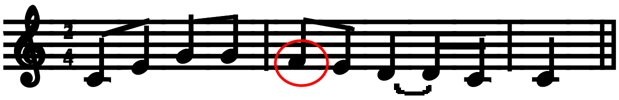

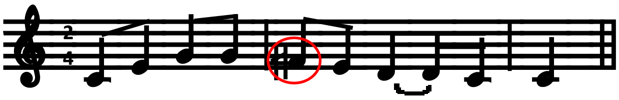

(b)

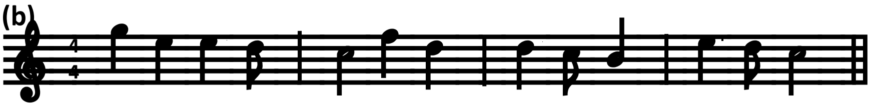

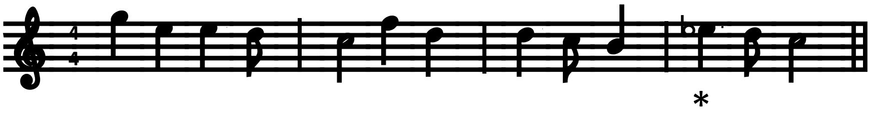

(c)

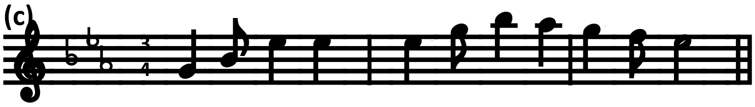

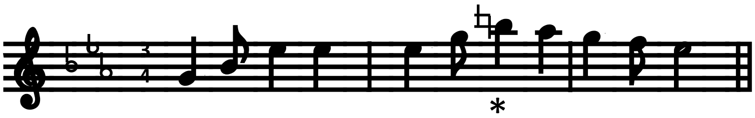

(d)

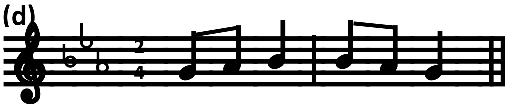

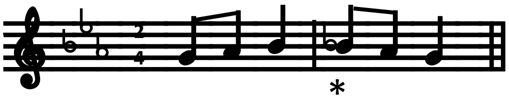

(e)

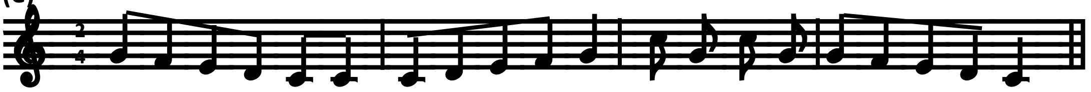

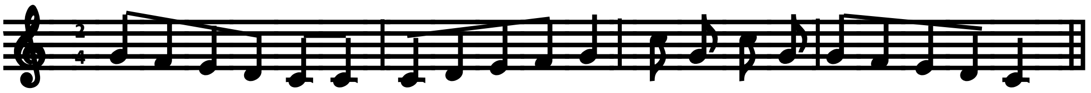

(f)

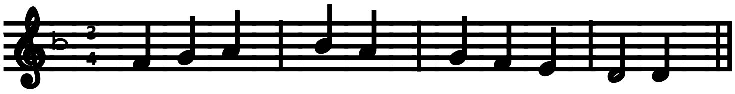

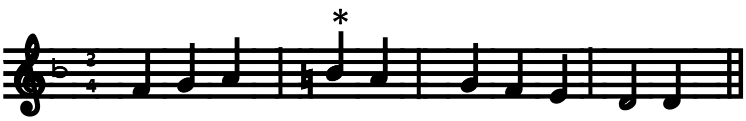

3. Interval Discrimination

(a)

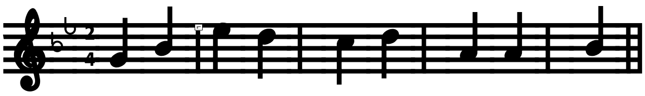

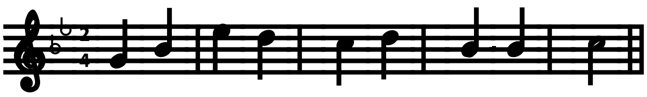

(b)

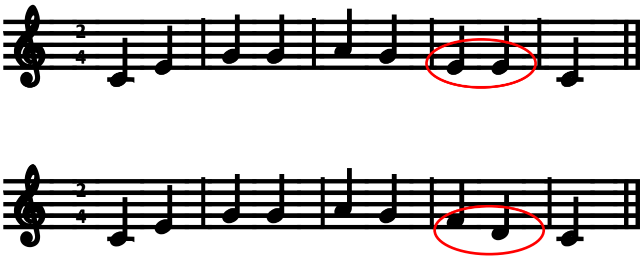

(c)

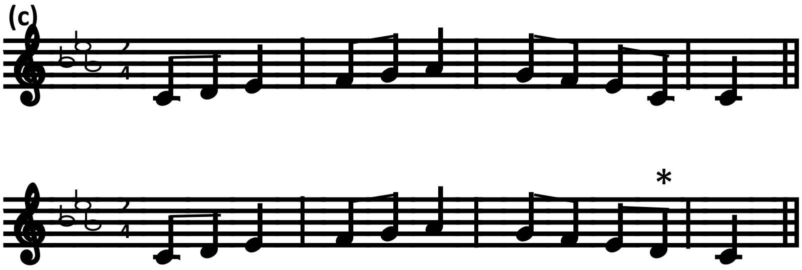

(d)

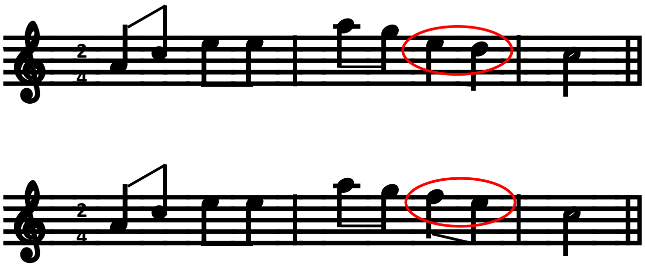

(e)

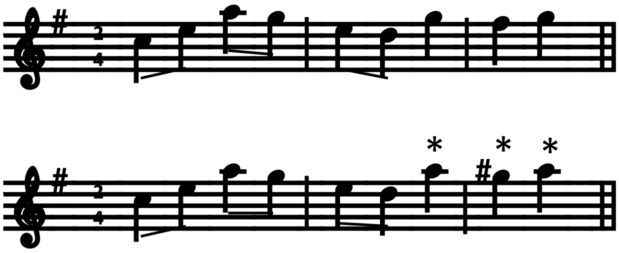

(f)

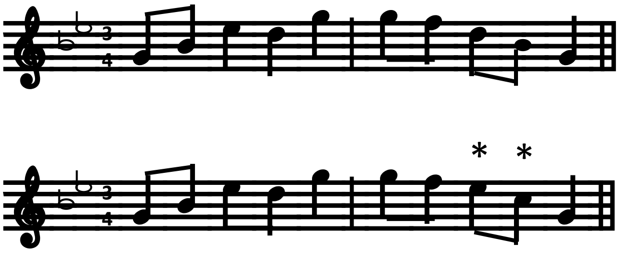

4. Memory

(a)

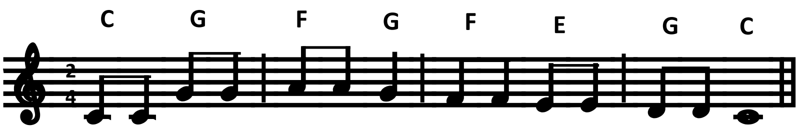

(b)

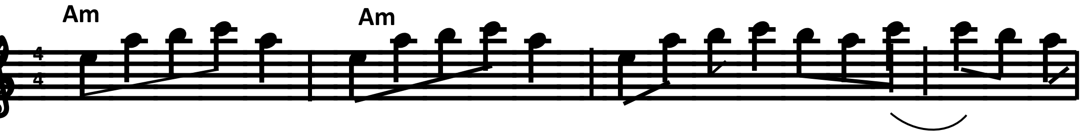

(c)

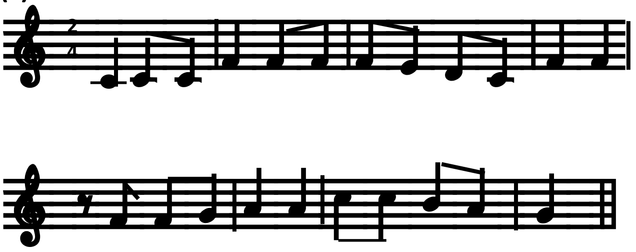

(d)

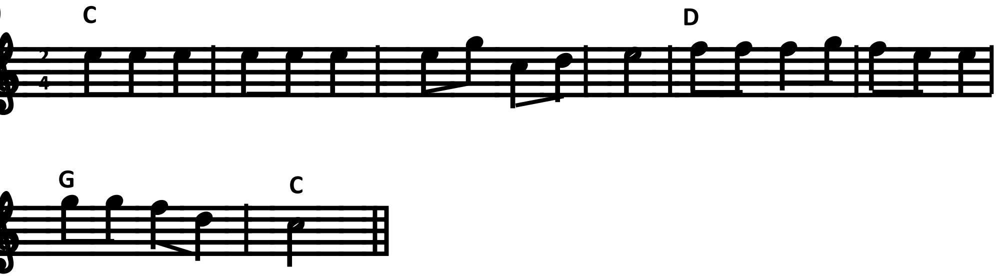

(e)

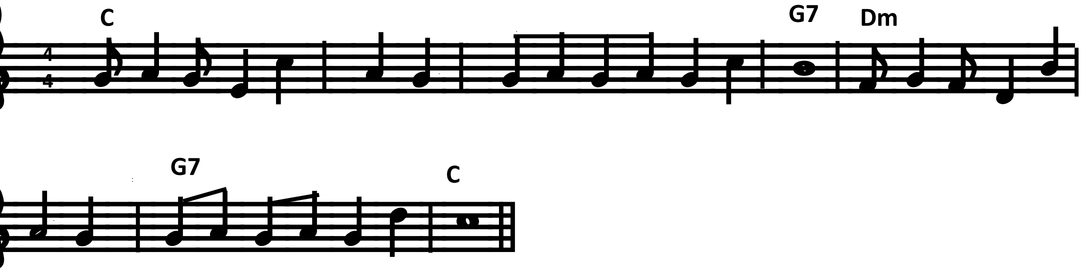

(f)

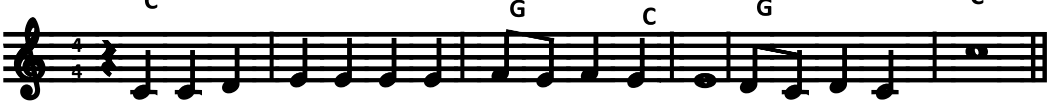

5. Rhythm

(a)

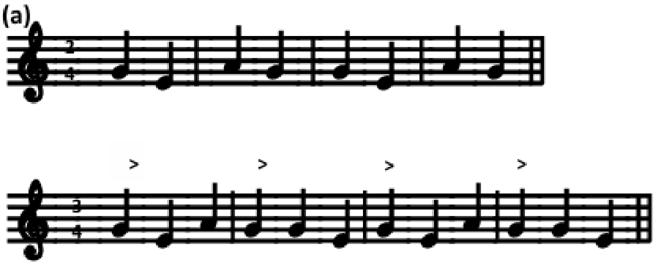

(b)

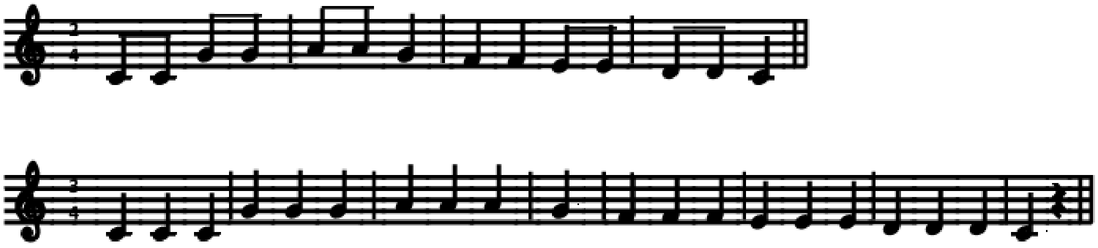

(c)

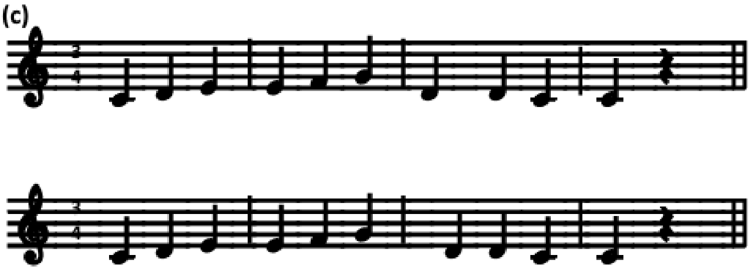

(d)

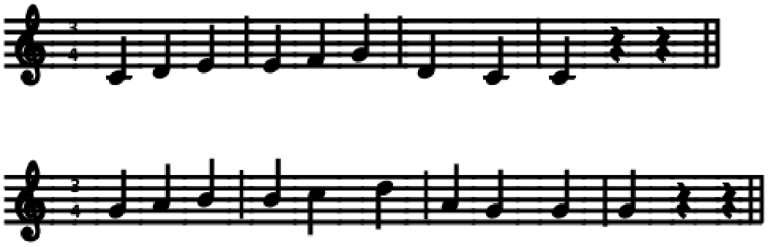

(e)

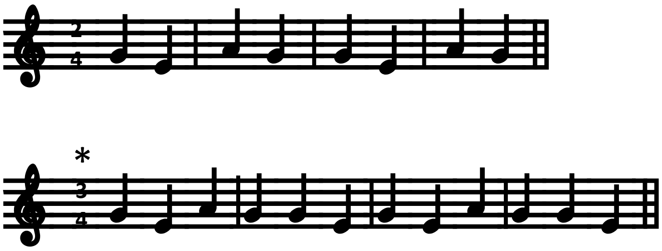

(f)

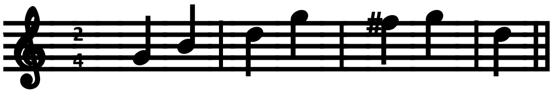

Δεν είναι δυνατή η εμφάνιση αυτής της εικόνας.

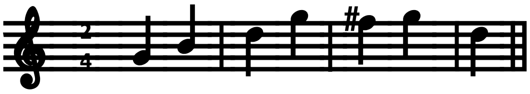

6. Intergration

(a)

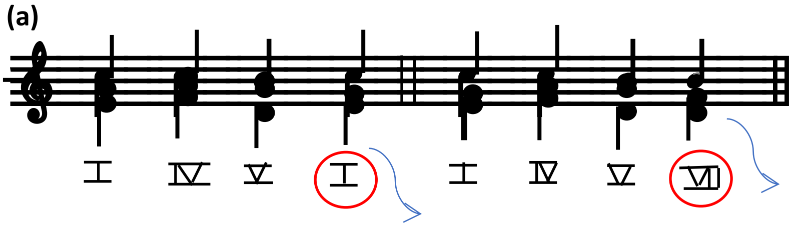

finished

Unfinished...

(b)

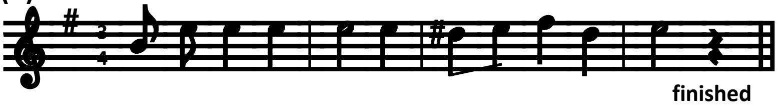

finished

(c)

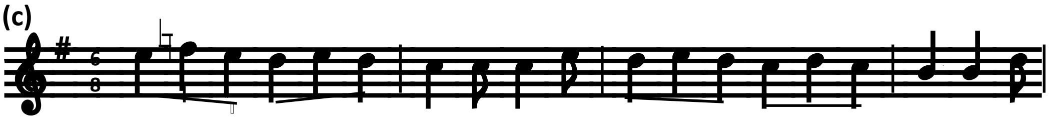

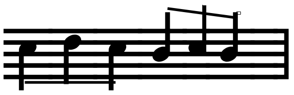

Unfinished

(d)

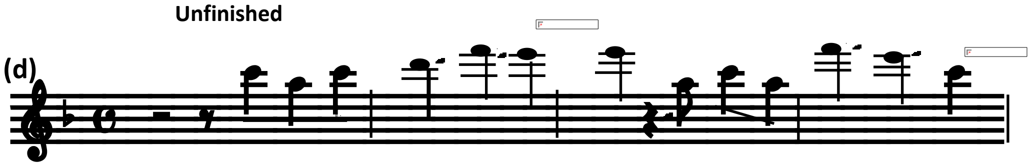

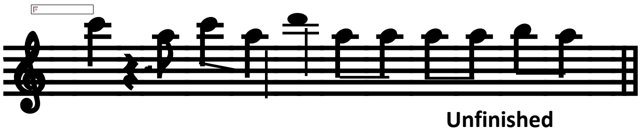

Unfinished

(e)

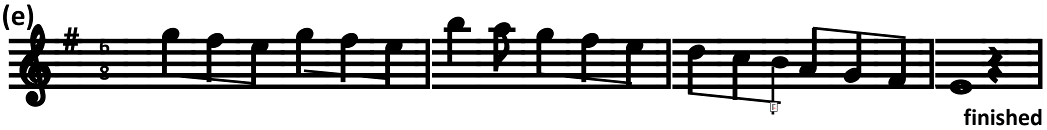

finished

(f)

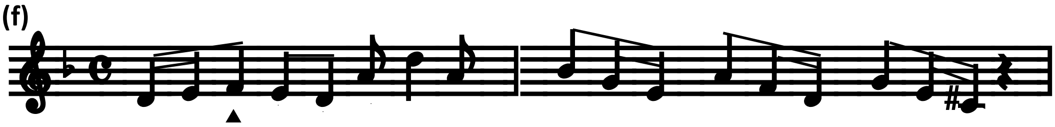

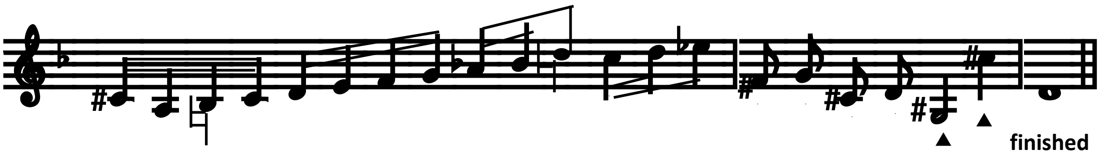

7. Emotion

(a)

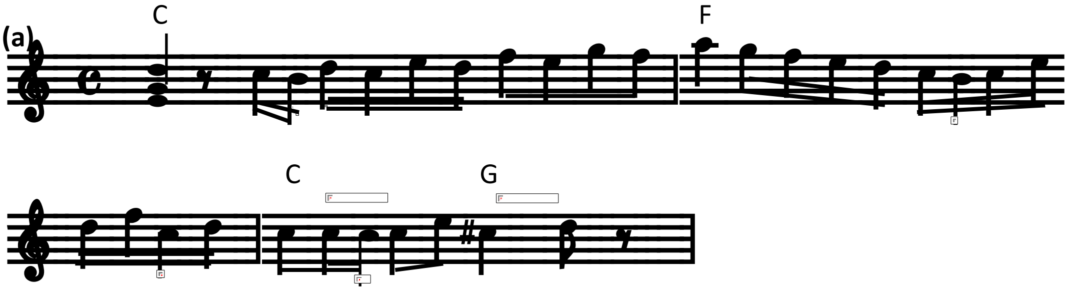

(b)

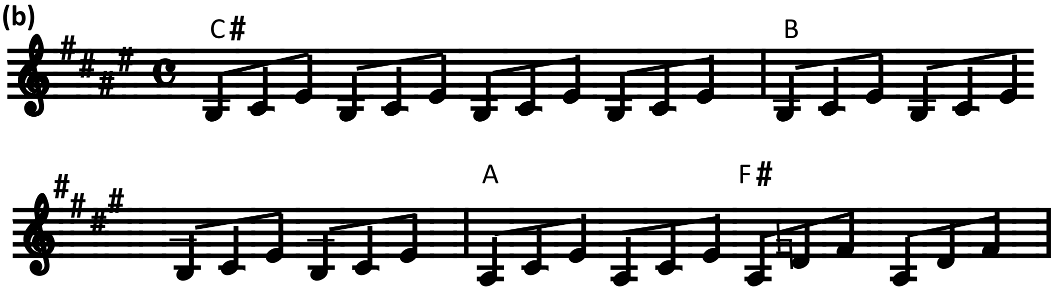

(c)

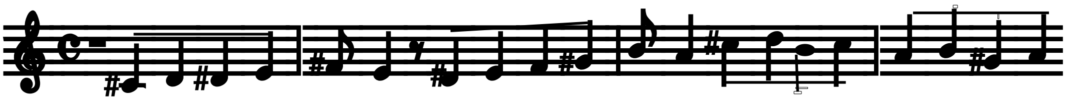

(d)

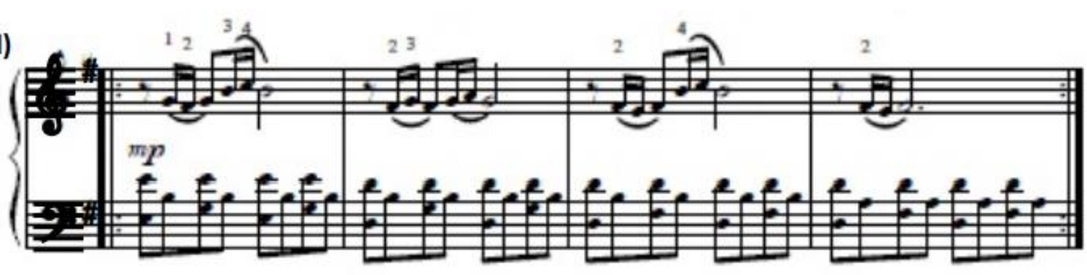

(e)

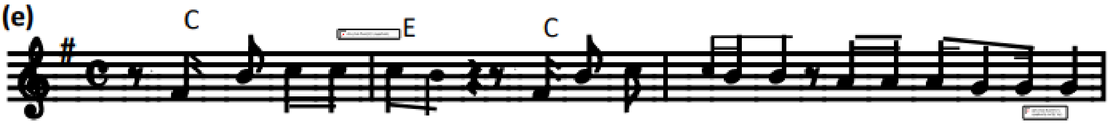

(f)

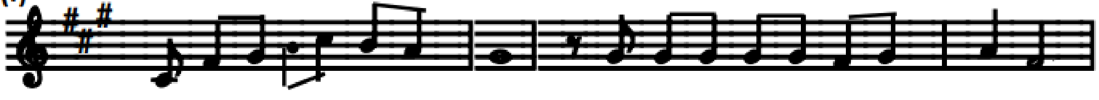

Supplement: Supplementary file 1 [file children-13-01156-s001.zip › File S1. Melodies of Amusia.pdf]
