# Supplementary material for: Musical Feature Processing and Learning Difficulties in Greek School-Aged Children: Piloting of a Screening Tool for Amusia
Source: Children (Basel). 2026 Aug 28;13(9):1156. doi: 10.3390/children13091156 (PMC13605103; doi:10.3390/children13091156)
Supplement: Supplementary file 1 [file children-13-01156-s001.zip › File S2. Questionnaire Responses.pdf]

QUESTIONNAIRE

Criteria for participation

- ✓ Normal hearing in both ears
- ✓ IQ above 85
- ✓ Absence of behavioral and emotional disorders

Student details

- **Age**

  Students : 6-7 , 7-8 , 8-9 , 9-10 , 10-11 , 11-12

  Adults : more than 18 years old
- **Music Education** : non | Until 2 years | more than 2 years
- **Normal audiometry** Yes , No
- **Gender** : Female | Male

Note

It is remarkable that the questionnaire is anonymous, optional and adheres to the code of ethics. All the above data will be used in this research for the application of appropriate tests in order to create a detection tool of amusia in general population.

SCREENING TOOL OF AMUSIA

RECORD RESPONSES

Intervals	Tone	Contour	Memory	Rhythm	Intergration	Emotion
/6	/6	/6	/6	/6	/6	/6

1. **DISSONANT INTERVALS** **Score**

Which sound is wrong?

1st pair of notes: 2nd pair is dissonant
2nd pair of notes: 1st
3rd pair of notes:1st

4th pair of notes: 2nd
5th pair of notes: 2nd
6th pair of notes: 2nd

/6

2. **OUT OF TONE** **Score**

Are the melodies identical or different?

1st Pair of Melodies: different

2nd Pair of Melodies: different

3rd Pair of Melodies: different

4th Pair of Melodies: different

5th Pair of Melodies: identical

6th Pair of Melodies: different

/6

3. **«CONTOUR»** **Score**

Are the melodies identical or different?

1st Pair of Melodies: different

2nt Pair of Melodies: different

3rd Pair of Melodies: different

4th Pair of Melodies: identical

5th Pair of Melodies: different

6th Pair of Melodies: different

/6

4. **«MEMORY»** **Score**

What little song does each melody remind you of?

1st melody: "twinkle little star"

2nd melody: "Bella Chiao,"

3rd melody: "The little rooster,"

4rh melody: "Jingle bells,"

5th melody: "Rudolph the red nose reindeer"

6th melody: "The drummer."

/6

5. **«Rythm»** **Score**

Are the melodies identical or different rhythmically?

1st Pair of Melodies: different

2nd Pair of Melodies: different

3rd Pair of Melodies: identical

4th Pair of Melodies: identical

5th Pair of Melodies: different

6th Pair of Melodies: identical

/6

6. **«INTERGRATION»** **Score**

Which melody is complete?

1st Pair of Melodies: finished-unfinished

2nt Melody: finished

3rd Melody: unfinished

4th Melody: unfinished

5th Melody: finished

6th Melody: finished

/6

7. «EMOTION» Score

Is the melody happy or sad?

1st Melody: happy

2nd Melodies: sad

3rd Melody: happy

4th Melody: sad

5th Melody: sad

6th Melody: sad

/6
